# Job Insecurity and Employees’ Taking Charge Behaviors: Testing a Moderated Mediation Model

**DOI:** 10.3390/ijerph19020696

**Published:** 2022-01-08

**Authors:** Fang Sun, Anqi Zheng, Junbang Lan

**Affiliations:** 1 Economics and Management School, Wuhan University, Wuhan 430072, China; sunfang@whu.edu.cn; 2 School of Electrical Engineering and Automation, Wuhan University, Wuhan 430072, China; anqizhengv@163.com; 3 School of Tourism Management, Sun Yat-sen University, Zhuhai 519000, China

**Keywords:** job insecurity, work engagement, taking charge, interactional justice, proactive behavior

## Abstract

Given the rapid changes in current technologies, business models, and work environments, organizations and managers increasingly rely on their employees’ proactive behaviors, such as taking charge, to gain competitive advantages. Taking charge involves a range of risky and future-oriented behaviors, and it requires employees to work hard to achieve them in the future. For employees with high job-insecurity, their job continuity in the future is threatened. Thus, they may not be willing to take risks to do additional work that is “future-oriented”. To our knowledge, the effect of job insecurity on employees’ taking charge has rarely been studied. As a result, the purpose of our study is to investigate whether, how, and when job insecurity will influence taking charge. Drawing on the conservation of resources theory and proactive motivation model, we develop a theoretical model. Moreover, we employed a multi-wave and multi-source survey to test our predictions. Based on the data from 194 full-time employees paired with their direct supervisors, the results provided consistent support for the proposed hypotheses. Specifically, the results indicate that job insecurity prohibits employees’ taking charge behaviors through deteriorating their work engagement. Furthermore, employees’ perception of interactional justice moderates the negative influence of job insecurity on their work engagement and, consequently, their taking charge behaviors. Theoretical and practical implications are discussed.

## 1. Introduction

Rapid changes in current technologies, business models, and work environments have restricted managers’ capabilities to deal with complex and dynamic issues [1,2]. To cope with these changes and maintain competitive advantages, increasing organizations are turning their attention to the employees because employees are the ones who are truly exposed to the “front line” and are most sensitive to changes when the company environment changes [3]. Organizations count on employees to proactively detect and solve problems to promote their effectiveness [1,2]. Given this trend among the practitioners, researchers have paid growing attention to proactive behaviors [2,4]. Among this research stream of proactive behaviors, taking charge has been studied the most [5,6]. Taking charge refers to “voluntary and constructive efforts, by individual employees, to effect organizationally functional change with respect to how work is executed within the contexts of their jobs, work units, or organizations” [7] (p. 403). Examples are actively modifying defective policies or improving production processes.

Research has revealed the antecedents of employees’ taking charge. For example, scholars have found that contextual factors such as output control [8], leader–member exchange [9], and team-member exchange [10] may affect taking charge. Individual differences factors such as curiosity [11], other-centered trait [12], and proactive personality [13] may also explain employees’ taking charge. Many valuable insights regarding taking charge have been provided to enrich our knowledge of it. However, our understanding of its antecedents is far from enough. Taking charge involves a range of risky and future-oriented behaviors [7]. Similar to other proactive behaviors, it requires employees to make predictions in advance based on the status quo, make plans, and work hard to achieve them in the future [14]. Hence, employees’ judgments about their future states can affect whether they choose to perform taking charge. Accordingly, we aim at exploring one of its important but neglected antecedents, namely job insecurity. Job insecurity is a perceptual phenomenon caused by potential threats to an employee’s future job security [15]. If employees are skeptical about the possibility of continuing working for their employer in the future, they may not be willing to take risks to do additional work that is “future-oriented”.

Drawing on the conservation of resources theory (COR) [16] and proactive motivation model (PMM) [17], we develop a theoretical model to investigate whether, how, and when employees’ job insecurity will influence their taking charge behaviors. According to COR theory, individuals tend to protect their current resources [16]. Moreover, those who already lack resources will be more likely to suffer loss spirals and thus may not be able to deal with future-loss threats [16]. Consequently, for those employees with low job-security, they have higher cost perception, less intrinsic motivation, and less activated positive emotion, which counter the motivational states that lead them to conduct proactive behaviors [17]. Conversely, work engagement means employees are active and committed [18], and it reflects employees’ proactive motivational states [18]. As a discretionary, challenging, and change-oriented behavior, taking charge can consume many resources [4]. Employees facing job insecurity lack resources and are anxious about future-loss threats [15]. Hence, we propose that they will pay fewer resources to their work and consequently conduct less taking charge.

**Figure 1:**
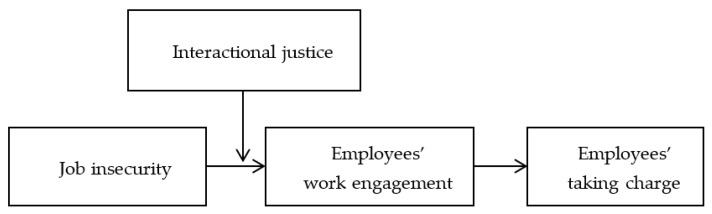
Overall research model.

However, based on COR theory, such frustrating processes can be buffered by gaining social resources in the workplace [19]. Similarly, Colquitt (2001) pointed out that if employees are treated well, they will feel better in the face of adverse situations [20]. Interactional justice reflects the quality of treatment employees feel during the process of communication [20]. This research speculates that interactional justice can alleviate the above negative effects. Specifically, we argue that interactional justice can buffer job insecurity’s harmful effects on taking charge through the mediation of work engagement. If employees feel their leaders’ and colleagues’ respect and care and have more comprehensive information about the decision-making of their organizations and teams, it can help them to gain social resources and then alleviate the stressful states induced by job insecurity. Figure 1 illustrates the overall research model.

Our theoretical model seeks to make several contributions to the existing literature. First, this research extends taking charge’s antecedents research by examining the influences of employees’ job insecurity on their taking charge. As noted, taking charge is resource-consuming and change-oriented behavior involving personal initiative [4]. In the exploration of the antecedents of taking charge, individual factors and situational factors have been unearthed systematically by existing studies. However, few studies have explored it from the perspective of personal resource constraints. Furthermore, limited research has examined taking charge’s antecedents through the lens of the judgment of the future states. Job insecurity reflects the lack of resources and fear of future loss [15]. Hence, investigating it as an antecedent can help to better understand predictors of taking charge, especially in the fast-developing and changing world. Second, we broaden the literature on employees’ taking charge by enriching the knowledge of its underlying mechanism. Based on the COR theory [16] and PMM [17], we seek to open the “black box” by examining the mediating mechanism linking employees’ job insecurity and their taking charge. Specifically, we argue that work engagement will mediate the above relationship.

Third, we contribute to the research on taking charge by broadening its boundary conditions. Previous research on taking charge mainly used individual and leadership characteristics as moderating factors. However, limited studies investigated its boundary conditions from the perspective of colleague interaction. Based on PMM [17], social contexts such as interactional justice can moderate the processes stimulating proactive behaviors. Thus, this research enriches the knowledge of taking charge by exploring whether the indirect effects of employees’ job insecurity on their taking charge through work engagement can be moderated by their perception of interactional justice. Moreover, by answering the question of whether we can design environments to share and foster resources through crossover processes among colleagues, our work has also made a contribution to the theory of COR [19]. Specifically, we speculate that the crossover of resources among colleagues works if organizations promote interactional justice.

Offering insights into the relationship between job insecurity and taking charge is also beneficial for practitioners. First, while the fast-changing work environment is making people around the world feel increasingly insecure about their jobs [21], organizations in this dynamic work environment increasingly expect their employees to take initiatives to gain long-term growth and success [1]. Through our findings of the harmful effects of job insecurity on employees’ taking charge behaviors, we encourage organizations to find solutions to alleviate employees’ perception of job insecurity. Second, interactional justice can mitigate the negative influences of job insecurity on employees’ work engagement and further on their taking charge behaviors. Hence, we suggest that if job insecurity is inevitable, organizations can mitigate its harmful effects on employees’ work behaviors by increasing interactional justice. We hope that our research will benefit the organizations and provide guidance for them to proactively deal with fast-changing situations.

## 2. Theory and Hypotheses Development

### 2.1. Job Insecurity and Taking Charge

A core point of COR is that individuals tend to conserve and increase resources [16]. When individuals feel that they are about to lose or valuable resources, they will feel threatened [16]. Accordingly, drawing on the COR theory, when employees face the loss of resources, they will first take measures to prevent further loss [16]. The more precious the resources, the stronger the employees’ intentions to avoid such losses [22]. Obviously, stable income and work are valuable resources that employees cherish [23]. The loss of these resources can influence employees’ attitudes and behaviors [16,24]. For example, employees will be more perfunctory in temporary work. According to Greenhalgh and Rosenblatt (1984), job insecurity reflects the degree of threat to job stability an employee feels [15]. It determines the stability of income and continuity of work. When the degree of job insecurity is high, it means that the resources employees value are threatened, such as a steady paycheck and job opportunity [23]. This threat will lead employees to pay more time and energy into protecting these cherished resources.

We propose that job insecurity can prevent employees from conducting taking charge behaviors for several reasons. First, employees experiencing job insecurity have strong intentions to protect their job opportunities, which are valuable resources to them [16,25]. Hence, they will pay more attention and energy to activities that help them maintain employment relationships [26]. For instance, employees may put effort into specific work to increase their performance in the short run. However, taking charge contains a set of change-oriented behaviors involving individual initiative, active observation, and dealing with setbacks [7]. It is regarded as future-oriented, resource-intensive, and highly risky [4]. Employees may worry about taking charge behaviors that can lead to poor performance in the short term or incur disgust from leaders and colleagues and further exacerbate the risk of losing their jobs. Consequently, when employees feel that their job insecurity is high, they are unlikely to conduct taking charge.

Second, the COR theory also holds that people strive to make resource surpluses to offset possible resource losses in the future [16,24]. If the degree of job insecurity is high, an employee may invest his or her valuable resources such as time and energy into activities with low risks, low cost, and high benefits when facing situations with multiple tasks or making choices among multiple goals [27]. For example, they may choose to practice impression management to establish good relationships with their leaders [28]. As a highly risky and high-cost behavior, taking charge will consume many employees’ resources in the future [29]. In addition, the benefits of taking charge are not easy to predict and obtain [7]. Hence, employees experiencing job insecurity are less likely to perform taking charge behaviors. Previous research suggested that job stressors (job insecurity is a type of stressor) could decrease employees’ proactive behavior [30]. Parker and his colleagues (2010) argued that employees with low job security have higher cost perception, less intrinsic motivation, and less activated positive emotion, which lead them to conduct less proactive behaviors [17]. Accordingly, we propose:

**Hypothesis** **1** **(H1).** *Job insecurity is negatively related to employees’ taking charge behaviors.*

### 2.2. Mediating Role of Work Engagement

Work engagement represents a positive and committed work state [18]. We speculate that job insecurity can reduce employees’ work engagement for several reasons based on the COR theory. First, maintaining a high degree of mental and physical investment in the workplace requires many resources [31]. For instance, at work, we not only have to invest in physical and mental labor but also face various complicated interpersonal relationships. However, employees will devote increasing resources to activities relating to maintaining job stability or seeking new job opportunities when they feel that the security of their job is not high. Therefore, the resources devoted to work will be reduced [32]. Second, the COR theory suggests that losing important resources will result in strained states [16]. It further reduces the amount of resources employees can devote to work. For example, previous studies found that job insecurity facilitated employees to lose their resources, feel anxious and focus less on work [33]. Obviously, such strained states can distract employees’ mental and physical resources. Therefore, we propose:

**Hypothesis** **2a** **(H2a).** *Job insecurity is negatively related to work engagement.*

In previous research, work engagement has been widely examined as the mediator linking work requirements, job resources, and routine behaviors [27]. Hence, we need to integrate the COR theory [16] with the PMM theory [17] to explain the mediating role of work engagement in linking employees’ job insecurity and taking charge behaviors. PMM theory suggests that people make decisions based on three fundamental motivational states before they perform a task [17]. The three proactive motivational states are “can do”, “reason to”, and “energized to” [17]. They are essential to stimulate individuals to conduct proactive behaviors such as taking charge [34]. “Can do” motivation refers to an individual’s self-efficacy reflecting one’s belief in successfully implementing proactive behaviors [17,34]. Employees can build their confidence in initiating proactive goals and further handling likely consequences only by engaging at work and investing many resources into work roles.

“Reason to” motivation explains why an individual chooses or insists on certain proactive goals [17,34]. A person inclines to behave proactively if he or she has strong intrinsic motivations or even experiences a sense of flow in doing their tasks [17]. Employees are highly engaged at work because they identify with their jobs [35,36]. Hence, they have reasons to engage in taking charge behaviors. The motive of “energized to” reflects the affect-related motivational states that can elicit individuals’ intentions to work hard to achieve proactive goals [17]. In particular, activated positive emotions are significant in inducing individuals to behave proactively [17]. Highly engaged employees put a large amount of vigor and emotional resources into work [35]. Hence, they have a high “energized to” incentive to conduct taking charge behaviors. To sum up and integrate Hypotheses 1 and 2a, we propose:

**Hypothesis** **2b** **(H2b).** *Work engagement is positively related to employees’ taking charge behaviors.*

**Hypothesis** **2c** **(H2c).** *Work engagement mediates the negative relationship between job insecurity and employees’ taking charge behaviors.*

### 2.3. Moderating Role of Interactional Justice

We have highlighted job insecurity predicted taking charge through the mediation of work engagement. However, this process can be alleviated. Based on the COR theory, obtaining personal, social, and material resources, such as good interpersonal relationships and support and care from colleagues, can help people adapt to the stressful situations resulting from the loss of resources [16]. Hence, once people have access to gain enough such resources, they can suffer less for losing their resources. Moreover, research has found that if employees are treated well, they will feel better in the face of adverse situations [20]. This research suggests that interactional justice can moderate job insecurity’s negative effect on employees’ work engagement for two reasons. First, based on the theory mentioned above, our research speculates if employees can obtain abundant social resources, the likelihood of resource loss caused by job insecurity threatening their intention to engage in work may be reduced. Typical social resources are social support from organizations, leaders, and colleagues [16,19]. According to Colquitt (2001), interactional justice is the degree of respect and care employees receive from their organization, leaders, and colleagues [20]. It is an important social support for them. Consequently, if an employee experiences strong interactional justice, the destructive impacts of job insecurity may be alleviated.

Second, Hobfoll and colleagues (2018) articulated that resources can be gained through the crossover processes among colleagues [19]. If an employee has a good relationship with his or her colleagues, then he or she has a higher probability of obtaining resources from those colleagues [19]. Similarly, some research found that good relationships between followers and their leaders can help them gain resources [37]. High interactional justice means that the relationship between the employee and colleagues is of high quality [20]. The resources are more likely to crossover from colleagues to employees who experience a high extent of interactional justice. Hence, the unfavorable impact on employees’ work engagement caused by job insecurity can be alleviated by interactional justice. This is because employees experiencing high interactional justice may accumulate resources through the crossover processes. To sum up, we hypothesize that:

**Hypothesis** **3a** **(H3a).** *Interactional justice moderates the negative relationship between job insecurity and employees’ work engagement. Specifically, such a negative relationship will be weaker when employees experience a high rather than a low extent of interactional justice.*

Integrating Hypothesis 2c and Hypothesis 3a, we propose:

**Hypothesis** **3b** **(H3b).** *Interactional justice moderates the mediating effect of job insecurity on employees’ taking charge behaviors through their work engagement. Specifically, such mediating effect will be weaker among employees experiencing a high rather than a low extent of interactional justice.*

## 3. Method

### 3.1. Participants and Procedure

We designed a multi-wave and multi-source survey to verify our hypotheses. Specifically, data were collected in eight Chinese companies across various industries such as communications services, financial services, and automobile manufacturing. These companies’ executive managers were Executive Master of Business Administration (EMBA) alumni of a business school the authors affiliated with and located in central China. With their approval, each companies’ HR manager assisted us in completing the data collection processes. It is worth highlighting that the executive managers and HR managers only acted as the “gatekeeper”. We randomly selected and invited tens of employees with their direct supervisors in each company to participate in our survey. In the invitation processes, we emphasized the complete confidentiality of this survey to them. Furthermore, we assured them 30 Chinese yuan (about 4.6 U.S. dollars) as a reward after they finished a three-wave survey. All questionnaires were labeled IDs to match the data.

In the first round, 236 employees evaluated their experiences of job insecurity, perceptions of interactional justice, and the control variables. After two weeks, 223 of the original 236 participants completed the second round of surveys evaluating their experiences of work engagement. After two weeks, again, 212 direct supervisors matched with the employees participated in the former two rounds of surveys completed the third round of surveys rating their employees’ taking charge behaviors. We excluded the questionnaires with incomplete responses. Therefore, our final sample consisted of 194 employees paired with their direct supervisors. The effective response rate was 82.20%. The final sample’s average age was 31.16 (SD = 7.11). A total of 42.8% were female, 72.2% of them had a bachelor’s degree or higher, and the average tenure was 5.62 years (SD = 5.29).

### 3.2. Measures

Following Brislin’s (1986) translation-back translation procedures, we translated all English scales into Chinese versions [38]. All items were measured using 5-point Likert scales (1 = strongly disagree, 5 = strongly agree) unless otherwise indicated.

Job insecurity. Employees rated their experiences of job insecurity at work. We used three items developed by Hellgren and Sverke (2003) [39]. A representative item was “There is a risk that I will have to leave my present job in the year to come”. Cronbach’s alpha was 0.79.

Interactional justice. We used a classic nine-item measurement from Colquitt (2001) [20]. A representative item was “My colleagues treated me with dignity” (1 = strongly disagree, 7 = strongly agree). Cronbach’s alpha was 0.95.

Work engagement. Employees assessed their work engagement by the 13 items from May and colleagues (2004) [40]. A representative item was “I exert a lot of energy performing my job”. Cronbach’s alpha was 0.74.

Taking charge. Each employee’s direct supervisor rated their subordinates’ taking charge behaviors. We used ten items from Morrison and Phelps (1999) to measure this variable [7]. A representative item was “This employee often tries to institute new work methods that are more effective for the company”. Cronbach’s alpha was 0.94.

Control variables. Our analyses identified demographic variables (e.g., gender, age, tenure, and education) as control variables. Moreover, based on the theoretical rationale of PMM [17], we speculated that procedural and distributive justice might influence employees’ “reason to” motivation to perform proactive behaviors. Meanwhile, empirical findings suggested that procedural justice and distributive justice could predict taking charge. For example, McAllister et al. (2007) found that procedural justice had a positive effect on taking charge [41]. Moon et al. (2008) found that distributive justice positively influenced taking charge [12]. To rule out their effects, we controlled for procedural justice and distributive justice in the analyses. Procedural justice and distributive justice were assessed using Colquitt’s (2001) classic scale [20]. A representative item of the procedural justice scale was “I’ve been able to express my views and feelings during the decision-making procedures” (1 = strongly disagree, 7 = strongly agree). Cronbach’s alpha was 0.91. A representative item of the distributive justice scale was “My outcome reflects the effort I have put into my work” (1 = strongly disagree, 7 = strongly agree). Cronbach’s alpha was 0.93.

## 4. Results

### 4.1. Preliminary Analysis

Table 1 reports the descriptive statistics and correlations of this research. A CFA with Amos 17.0 software was conducted to examine the discriminant validity of our latent variables. Recommended by previous research [42], we applied the item parceling procedures before running the CFAs. The proposed six-factor model showed good fit (*χ*^2^ = 725.88, *df* = 362, CFI = 0.91, IFI = 0.91, RMSEA = 0.07). Relative to the alternative nested models, the above results indicated that our focal variables had appropriate distinctiveness. For example, a five-factor model that constrained procedural justice and distributive justice to load on the same factor showed poorer fit (CFI = 0.88, IFI = 0.88, RMSEA = 0.08, Δ*χ*^2^ = 119.66, Δ*df* = 5, *p* < 0.001).

### 4.2. Hypotheses Testing

We conducted hierarchical regression analyses using SPSS 23.0 (IBM, New York, USA) to verify our hypotheses. All the predicting variables (except the dummies) were standardized, transformed to make it easier to interpret the findings. Table 2 presents the results of the hierarchical regression analyses. As shown in Model 4, job insecurity had a negative effect on employees’ taking charge (*B* = −0.46, *p* < 0.001). The results in Model 2 indicated that job insecurity had a negative effect on employees’ work engagement (*B* = −0.18, *p* < 0.001). The results in Model 6 showed that employees’ work engagement had a positive effect on their taking charge (*B* = 0.22, *p* < 0.001) after controlling for job insecurity. Thus, hypotheses 1, 2a, and 2b were supported.

Following recommendations from Baron and Kenny (1986) [43], we continued to test the mediating effect of work engagement in linking job insecurity and taking charge (Hypothesis 2c). Based on the results supporting Hypothesis 1, 2a, and 2c, and the diminished coefficient in Model 6 (*B* = −0.39, *p* < 0.001), all the requirements for testing mediating effects were fulfilled. Hence, Hypothesis 2c was supported. Moreover, we conducted a bootstrapping procedure to further directly test this mediating effect using RMediation [44]. The results showed that the negative mediating effect of job insecurity on employees’ taking charge through their work engagement is significant (indirect effect = −0.04, 95% CI = [−0.06, −0.02]). Accordingly, Hypothesis 2c was further supported.

**Figure 2:**
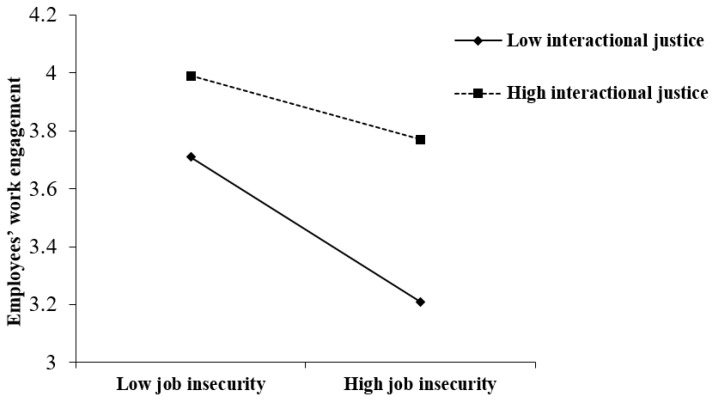
The moderating effect of interactional justice on the relationship between job insecurity and employees’ work engagement.

In addition, the results of Model 3 suggested interactional justice interacted with job insecurity to predict employees’ work engagement (*B* = 0.07, *p* < 0.05). We also followed Aiken and West’s (1991) recommendation [45] and plotted the moderating effect in Figure 2. Supporting Hypothesis 3a, the results of simple slope analyses showed job insecurity was more negatively related to employees’ work engagement when interactional justice was low (simple slope = −0.25, *p* < 0.001) than when interactional justice was high (simple slope = −0.11, *p* < 0.05).

To assess the moderated mediation effect (Hypothesis 3b), we used the Monte Carlo technique in R software following Preacher and Selig’s (2012) recommendation [46]. The results showed when interactional justice was low (indirect effect = −0.06, 95% CI = [−0.09, −0.03]) than when interactional justice was high (indirect effect = −0.02, 95% CI = [−0.04, −0.01]; difference = 0.04, 95% CI = [0.01, 0.06]), the indirect effect of job insecurity on employees’ taking charge via their work engagement was more significant. Overall, Hypothesis 3b was supported.

## 5. Discussion

We integrate the theory of COR and PMM to explore whether, how, and when job insecurity can decrease employees’ taking charge behaviors. Specifically, the results indicate that job insecurity hurts employees’ taking charge behaviors. Employees’ work engagement mediates this effect. Moreover, employees’ perception of interactional justice moderates job insecurity’s negative influence on their work engagement. Furthermore, the interactional justice moderates the indirect effect of job insecurity on their taking charge behaviors through work engagement. We next discuss the theoretical and practical implications and limitations of this research.

### 5.1. Theoretical Implications

Our research has several theoretical implications. First, we contribute to the literature on the outcomes of job insecurity and the antecedents of proactive behaviors. By exploring how employees’ concerns about job security have a consequence for their taking charge, we have revealed the critical factor of job insecurity. Increasing research has found some important antecedents, such as leadership (e.g., empowering leadership) [47,48], individual traits (e.g., curiosity; proactive personality) [11,13]. These studies provide us with many valuable insights regarding taking charge. However, taking charge behaviors are risky and resource-intensive [4]. Few studies have examined whether social context with resources constraint can prohibit employees from conducting taking charge behaviors. Job insecurity reflects employees’ perception of the organizational underemployment policies which foreshadows the lack of resources [15]. Hence, by investigating its negative effect on employee’ taking charge behaviors, we can broaden the research on proactive behaviors such as taking charge by suggesting that social context lacking resources will inhibit employees’ proactive behaviors. To our knowledge, no research has examined the antecedents of taking charge through the lens of the judgment of future states. Job insecurity reflects the fear of future resources loss [15]. Accordingly, examining its influence on taking charge can enrich our understanding of the antecedents of proactive behaviors by, to some extent, incorporating the dimension of “time.” Our findings are consistent with the previous literature that explored the impact of job insecurity on proactive behaviors. For example, Koen and van Bezouuw (2021) demonstrated that people’s worry about job security undermined their proactive career behavior [49]. Probst et al. (2007) found that job insecurity had a detrimental impact on employees’ creativity [50]. Yao et al. (2021) found that job insecurity could negatively impact employees’ proactive behavior [51].

We also contribute to research on proactive behaviors by enriching the knowledge of how social context influences taking charge behaviors. As noted, based on the theory of COR [16] and PMM [17], this study opens the “black box” by examining how the relationship between employees’ job insecurity and their taking charge is affected by work engagement. In accordance with the theoretical framework of Cai et al. (2019) [33], we demonstrate that the resource constraint perception caused by social context (i.e., job insecurity in this study) can weaken employees’ intentions to behave proactively via harming their “can do”, “reason to”, and “energized to” motivations (integrated as the experience of work engagement in this study). Hence, we answer the call for examining whether and how employees’ proactive behavior is hindered by social context. Our research findings enrich recent studies investigating the relationship and underlying mechanism between social context factors and proactive behaviors. For example, Liu et al. (2021) found that perceived crisis strength in COVID-19 reduced an employee’s taking charge behavior by reducing their work engagement [52]. Li et al. (2021) demonstrated that the negative impact of authoritarian leadership and abusive supervision on employees’ proactive behavior was mediated by their feelings of powerlessness [53].

Finally, we broaden the theory on proactive behaviors by enriching its boundary conditions. Previous studies are concerned mainly about the buffer factors of individual and leadership characteristics [33]. Nevertheless, the moderating role of other factors of social context was neglected. Thus, this research answers Cai, Parker, and colleagues’ (2019) call to consider the interaction of social context elements [33]. Specifically, our research highlights the harmful influence of employees’ job insecurity on their taking charge through work engagement can be buffered by their interactional justice. Moreover, this research answers Hobfoll and colleagues’ (2018) call to examine whether we can design environments to share and foster resources through crossover processes among colleagues by finding the buffering effects of interactional justice [19]. Our findings enrich current research that explored social context factors as the moderators. For example, Liu et al. (2021) demonstrated that employees’ perception of meaningful work could moderate the negative effect of perceived COVID-19 crisis strength on taking charge [52]. Tian et al. (2021) found that work group status diversity could moderate the relationship between job crafting and employee creativity [54].

### 5.2. Practical Implications

The organizations can benefit from our research findings by drawing insights into when the organization can hurt employees’ intentions to take initiatives and behave proactively. Specifically, we suggest that organizations should increase job security because employees will seek to conserve their resources, hold on to their energy, and avoid conducting proactive behaviors that are risky and resource-consuming when they experience job insecurity. Moreover, when job insecurity is unavoidable, organizations can alleviate its harmful effects on proactive behavior by increasing interactional justice. For example, organizations should advocate that the managers show more respect and care to their employees, keep the transparency of their decision-making processes, establish open communication channels, and try to achieve fair procedures.

### 5.3. Limitations and Future Research

Our research has some limitations. First, although our study employed a multi-wave and multi-source survey to minimize the common method bias, this design still prevents us from making causal inferences. Hence, future studies can consider longitudinal or experimental studies to further test our model. Second, although based on the literature on work engagement, we believe it can appropriately reflect employees’ proactive motivation (i.e., “can do”, “reason to”, and “energized to”). However, it threatens the possibility to compare and analyze the nuanced differences of the three different proactive motivations in linking job insecurity and taking charge. We encourage future research can further explore the mediating effects of the three proactive motivations, respectively.

## 6. Conclusions

This study explored whether, when, and how employees’ job insecurity could influence their taking charge behaviors. Based on the multi-source and multi-wave data from 194 full-time employees paired with their direct supervisors, we found that employees’ job insecurity negatively affected their taking charge behaviors, indicating that employees with high job insecurity perceptions were less likely to conduct proactive behaviors such as taking charge. Moreover, the results showed that employees’ work engagement was the underlying mechanism linking job insecurity and taking charge behaviors. In addition, we also found that employees’ perception of interactional justice was the boundary condition of the above relationships. Specifically, in the context with low rather than high interactional justice, employees’ feeling of job insecurity was more likely to deteriorate their work engagement and consequently decrease their taking charge behaviors. In sum, we broadened the literature of taking charge by examining job insecurity as a critical predictor.

## Figures and Tables

**Table 1:** Descriptive statistics and correlations of variables.

Variables	Mean	SD	1	2	3	4	5	6	7	8	9	10
1. Gender	0.57	0.50	-									
2. Educational level	1.73	0.62	−0.17 *	-								
3. Age	31.16	7.11	0.14	−0.08	-							
4. Tenure	5.62	5.29	−0.00	0.01	0.74 **	-						
5. Procedural justice	4.84	1.08	0.01	0.18 *	−0.05	−0.02	(0.91)					
6. Distributive justice	5.04	1.23	0.01	0.10	−0.08	−0.02	0.47 *	(0.93)				
7. Job insecurity	2.43	0.93	0.18 *	−0.07	0.05	−0.06	−0.15 *	−0.14 *	(0.79)			
8. Work engagement	3.67	0.51	−0.12	0.11	−0.06	−0.04	0.23 **	0.19 **	−0.37 **	(0.74)		
9. Interactional justice	5.27	1.07	−0.07	0.17 *	−0.03	−0.02	0.37 **	0.35 **	−0.29 **	0.33 **	(0.95)	
10. Taking charge	3.46	0.79	0.20 **	0.15 *	−0.10	0.01	0.18 *	0.22 **	−0.36 **	0.36 **	0.38 **	(0.94)

Note. *n* = 194; Reliabilities are in parentheses on the diagonal; SD is short for standard deviation; * *p* < 0.05; ** *p* < 0.01.

**Table 2:** The results of hierarchical regression analyses.

Variables	Employees’ Work Engagement			Employees’ Taking Charge		
	Model 1	Model 2	Model 3	Model 4	Model 5	Model 6
Intercept	3.73 ***	3.69 ***	3.67 ***	3.61 ***	3.52 ***	3.51 ***
Control variables						
Gender	−0.11	−0.03	−0.03	−0.26 *	−0.10	−0.09
Educational level	0.03	0.00	0.00	0.07	0.05	0.05
Age	0.00	0.01	0.01	−0.11	−0.06	−0.06
Tenure	−0.02	−0.03	−0.03	0.09	0.03	0.04
Procedural justice	0.09	0.03	0.03	0.01	−0.07	−0.09
Distributive justice	0.02	−0.02	−0.04	0.15	0.10	0.11
Independent variable						
Job insecurity		−0.18 ***	−0.18 ***		−0.46 ***	−0.39 ***
Mediator						
Employees’ work engagement						0.22 ***
Moderator						
Interactional justice		0.22 ***	0.21 ***		0.15 **	0.06
Interaction term						
Job insecurity × Interactional justice			0.07 *			
R^2^	0.07	0.39	0.41	0.11	0.50	0.55
ΔR^2^		0.32 ***	0.02 *		0.39 ***	0.05 *

Note. *n* = 194; * *p* < 0.05; ** *p* < 0.01; *** *p* < 0.001.

## Data Availability

The data presented in this study are available on request from the corresponding author. The data are not publicly available due to respondents’ privacy.

## References

[1] Zhang M.J., Law K.S., Wang L. (2021). The risks and benefits of initiating change at work: Social consequences for proactive employees who take charge. Pers. Psychol..

[2] Schilpzand P., Houston L., Cho J. (2018). Not Too Tired to be Proactive: Daily Empowering Leadership Spurs Next-morning Employee Proactivity as Moderated by Nightly Sleep Quality. Acad. Manag. J..

[3] Cai Z., Huo Y., Lan J., Chen Z., Lam W. (2019). When Do Frontline Hospitality Employees Take Charge? Prosocial Motivation, Taking Charge, and Job Performance: The Moderating Role of Job Autonomy. Cornell Hosp. Q..

[4] Ouyang K., Cheng B.H., Lam W., Parker S.K. (2019). Enjoy your evening, be proactive tomorrow: How off-job experiences shape daily proactivity. J. Appl. Psychol..

[5] Burnett M.F., Chiaburu D.S., Shapiro D.L., Li N. (2015). Revisiting how and when perceived organizational support enhances taking charge: An inverted U-shaped perspective. J. Manag..

[6] Li N., Chiaburu D.S., Kirkman B.L., Xie Z. (2013). Spotlight on the Followers: An Examination of Moderators of Relationships between Transformational Leadership and Subordinates’ Citizenship and Taking Charge. Pers. Psychol..

[7] Morrison E.W., Phelps C.C. (1999). Taking charge at work: Extrarole efforts to initiate workplace change. Acad. Manag. J..

[8] Chiaburu D.S., Baker V.L. (2006). Extra-role behaviors challenging the status-quo: Validity and antecedents of taking charge behaviors. J. Manag. Psychol..

[9] Kim T.Y., Liu Z., Diefendorff J.M. (2015). Leader–member exchange and job performance: The effects of taking charge and organizational tenure. J. Organ. Behav..

[10] Love M.S., Dustin S.L. (2014). An investigation of coworker relationships and psychological collectivism on employee propensity to take charge. Int. J. Hum. Resour. Manag..

[11] Harrison S.H., Sluss D.M., Ashforth B.E. (2011). Curiosity adapted the cat: The role of trait curiosity in newcomer adaptation. J. Appl. Psychol..

[12] Moon H., Kamdar D., Mayer D.M., Takeuchi R. (2008). Me or we? The role of personality and justice as other-centered antecedents to innovative citizenship behaviors within organizations. J. Appl. Psychol..

[13] Parker S.K., Collins C.G. (2010). Taking stock: Integrating and differentiating multiple proactive behaviors. J. Manag..

[14] Grant A.M., Ashford S.J. (2008). The dynamics of proactivity at work. Res. Organ. Behav..

[15] Greenhalgh L., Rosenblatt Z. (1984). Job insecurity: Toward conceptual clarity. Acad. Manag. Rev..

[16] Hobfoll S.E. (1989). Conservation of resources-A new attempt at conceptualizing stress. Am. Psychol..

[17] Parker S.K., Bindl U.K., Strauss K. (2010). Making Things Happen: A Model of Proactive Motivation. J. Manag..

[18] Schaufeli W.B., Salanova M., González-romá V., Bakker A.B. (2002). The Measurement of Engagement and Burnout: A Two Sample Confirmatory Factor Analytic Approach. J. Happiness Stud..

[19] Hobfoll S.E., Halbesleben J., Neveu J.-P., Westman M. (2018). Conservation of Resources in the Organizational Context: The Reality of Resources and Their Consequences. Annu. Rev. Organ. Psych..

[20] Colquitt J.A. (2001). On the dimensionality of organizational justice: A construct validation of a measure. J. Appl. Psychol..

[21] Patnaik S., Mishra U.S., Mishra B.B. (2021). Can psychological capital reduce stress and job insecurity? An experimental examination with indian evidence. Asia Pac. J. Manag..

[22] Rappaport J. (1981). In praise of paradox: A social policy of empowerment over prevention. Am. J. Community Psychol..

[23] Sverke M., Hellgren J., Naswell K. (2002). No security: A meta-analysis and review of job insecurity and its consequences. J. Occup. Health Psychol..

[24] Hobfoll S.E. (2001). The Influence of Culture, Community, and the Nested-Self in the Stress Process: Advancing Conservation of Resources Theory. Appl. Psychol.-Int. Rev..

[25] Shoss M.K., Brummel B.J., Probst T.M., Jiang L. (2020). The Joint Importance of Secure and Satisfying Work: Insights from Three Studies. J. Bus. Psychol..

[26] Schumacher D., Schreurs B., De Cuyper N., Grosemans I. (2021). The ups and downs of felt job insecurity and job performance: The moderating role of informational justice. Work Stress.

[27] Shin Y., Hur W.M. (2021). When do job-insecure employees keep performing well? The buffering roles of help and prosocial motivation in the relationship between job insecurity, work engagement, and job performance. J. Bus. Psychol..

[28] Huang G.H., Zhao H.H., Niu X.Y., Ashford S.J., Lee C. (2013). Reducing job insecurity and increasing performance ratings: Does impression management matter?. J. Appl. Psychol..

[29] Strauss K., Parker S.K., O’Shea D. (2017). When does proactivity have a cost? Motivation at work moderates the effects of proactive work behavior on employee job strain. J. Vocat. Behav..

[30] Fritz C., Sonnentag S. (2007). Antecedents of day-level proactive behavior: A look at job stressors and positive affect during the workday. J. Manag..

[31] Bakker A.B., Schaufeli W.B., Leiter M.P., Taris T.W. (2008). Work engagement: An emerging concept in occupational health psychology. Work Stress.

[32] Schreurs B., Hetty van Emmerik I., Günter H., Germeys F. (2012). A weekly diary study on the buffering role of social support in the relationship between job insecurity and employee performance. Hum. Resour. Manag..

[33] Wang H.J., Lu C.Q., Siu O.L. (2015). Job insecurity and performance: The moderating role of organizational justice and the mediating role of work engagement. J. Appl. Psychol..

[34] Cai Z., Parker S.K., Chen Z., Lam W. (2019). How does the social context fuel the proactive fire? A multilevel review and theoretical synthesis. J. Organ. Behav..

[35] Bakker A.B., Demerouti E., Sanz-Vergel A.I. (2014). Burnout and work engagement: The JD–R approach. Annu. Rev. Organ. Psych..

[36] Kahn W.A. (1990). Psychological conditions of personal engagement and disengagement at work. Acad. Manag. J..

[37] Breevaart K., Bakker A., Hetland J., Demerouti E., Olsen O., Espevik R. (2014). Daily transactional and transformational leadership and daily employee engagement. J. Occup. Organ. Psychol..

[38] Brislin R.W., Lonner W., Berry J. (1986). The wording and translation of research instrument. Field Methods in Cross-Cultural Research.

[39] Hellgren J., Sverke M. (2003). Does job insecurity lead to impaired well-being or vice versa? Estimation of cross-lagged effects using latent variable modelling. J. Organ. Behav..

[40] May D.R., Gilson R.L., Harter L.M. (2004). The psychological conditions of meaningfulness, safety and availability and the engagement of the human spirit at work. J. Occup. Organ. Psychol..

[41] McAllister D.J., Kamdar D., Morrison E.W., Turban D.B. (2007). Disentangling role perceptions: How perceived role breadth, discretion, instrumentality, and efficacy relate to helping and taking charge. J. Appl. Psychol..

[42] Hau K.-T., Marsh H.W. (2004). The use of item parcels in structural equation modelling: Non-normal data and small sample sizes. Br. J. Math. Stat. Psychol..

[43] Baron R.M., Kenny D.A. (1986). The moderator-mediator variable distinction in social psychological research: Conceptual, strategic, and statistical considerations. J. Pers. Soc. Psychol..

[44] Tofighi D., MacKinnon D.P. (2011). RMediation: An R package for mediation analysis confidence intervals. Behav. Res. Methods.

[45] Aiken L.S., West S.G. (1991). Multiple Regression: Testing and Interpreting Interactions.

[46] Preacher K.J., Selig J.P. (2012). Advantages of Monte Carlo Confidence Intervals for Indirect Effects. Commun. Methods Meas..

[47] Li N., Chiaburu D.S., Kirkman B.L. (2017). Cross-Level Influences of Empowering Leadership on Citizenship Behavior: Organizational Support Climate as a Double-Edged Sword. J. Manag..

[48] Li S.L., He W., Yam K.C., Long L.R. (2015). When and why empowering leadership increases followers’ taking charge: A multilevel examination in China. Asia Pac. J. Manag..

[49] Koen J., van Bezouw M.J. (2021). Acting Proactively to Manage Job Insecurity: How Worrying About the Future of One’s Job May Obstruct Future-Focused Thinking and Behavior. Front. Psychol..

[50] Probst T.M., Stewart S.M., Gruys M.L., Tierney B.W. (2007). Productivity, counterproductivity and creativity: The ups and downs of job insecurity. J. Occup. Organ. Psychol..

[51] Yao X., Li M., Zhang H.Q. (2021). Suffering Job Insecurity: Will the Employees Take the Proactive Behavior or Not?. Front. Psychol..

[52] Liu D., Chen Y., Li N. (2021). Tackling the negative impact of COVID-19 on work engagement and taking charge: A multi-study investigation of frontline health workers. J. Appl. Psychol..

[53] Li R., Chen Z., Zhang H., Luo J. (2021). How Do Authoritarian Leadership and Abusive Supervision Jointly Thwart Follower Proactivity? A Social Control Perspective. J. Manag..

[54] Tian W., Wang H., Rispens S. (2021). How and When Job Crafting Relates to Employee Creativity: The Important Roles of Work Engagement and Perceived Work Group Status Diversity. Int. J. Environ. Res. Public Health.

